# Cardiac Cachexia: Perspectives for Prevention and Treatment

**DOI:** 10.5935/abc.20160142

**Published:** 2017-01

**Authors:** Marina Politi Okoshi, Rafael Verardino Capalbo, Fernando G Romeiro, Katashi Okoshi

**Affiliations:** Departamento de Clínica Médica - Faculdade de Medicina de Botucatu - Universidade Estadual Paulista - UNESP, São Paulo, SP - Brazil

**Keywords:** Heart Failure, Muscle Wasting, Physical Exercise, Prognosis, Nutrition, Anemia

## Abstract

Cachexia is a prevalent pathological condition associated with chronic heart failure. Its occurrence predicts increased morbidity and mortality independent of important clinical variables such as age, ventricular function, or heart failure functional class. The clinical consequences of cachexia are dependent on both weight loss and systemic inflammation, which accompany cachexia development. Skeletal muscle wasting is an important component of cachexia; it often precedes cachexia development and predicts poor outcome in heart failure. Cachexia clinically affects several organs and systems. It is a multifactorial condition where underlying pathophysiological mechanisms are not completely understood making it difficult to develop specific prevention and treatment therapies. Preventive strategies have largely focused on muscle mass preservation. Different treatment options have been described, mostly in small clinical studies or experimental settings. These include nutritional support, neurohormonal blockade, reducing intestinal bacterial translocation, anemia and iron deficiency treatment, appetite stimulants, immunomodulatory agents, anabolic hormones, and physical exercise regimens. Currently, nonpharmacological therapy such as nutritional support and physical exercise are considered central to cachexia prevention and treatment.

## Introduction

Heart failure is an important public health issue due to a high prevalence, severity of clinical manifestations and poor prognosis. Statistical data from the United States estimate that 5.7 million Americans over 20 years of age have heart failure; this is expected to increase by approximately 46% between 2012 and 2030, resulting in over 8 million adults with heart failure.^1^

Heart failure is caused by structural and functional abnormalities in the heart leading to impaired ventricular ejection and/or filling capacity. In Brazil, the main causes of heart failure are myocardial ischemia, systemic arterial hypertension, dilated cardiomyopathy and Chagas' disease, and valve disease.^2^ Following cardiac injury, the ensuing molecular, structural, and functional ventricular changes are known as cardiac remodeling. This process is accompanied by cardiac and systemic neurohormonal and inflammatory activation, which adversely affects the heart in a vicious cycle and jeopardizes different organs and systems.^3^ In recent decades, it has become clear that pathological changes involve not only the cardiovascular system, but also the renal, neuroendocrinological, immunological, hematologic, gastrointestinal, and musculoskeletal systems, as well as the nutritional status. Currently, experimental and clinical studies have focused on the physiopathology of heart failure-related systemic complications in order to establish treatments to improve quality of life and increase survival.

Cachexia is a prevalent and important pathological condition associated with chronic heart failure. Its occurrence predicts reduced survival, independent of relevant variables such as age, heart failure functional class, ejection fraction, and physical capacity.^4^ We evaluate studies on heart failure-induced cachexia and discuss different therapies for its prevention and treatment.

## Cardiac cachexia definition

Cachexia has been defined as at least 5% edema-free body weight loss in the previous 12 months (or a body mass index < 20 kg/m^2^) in patients with chronic illness and at least three of the following clinical or laboratory criteria: decreased muscle strength, fatigue, anorexia, low fat-free mass index and abnormal biochemistry characterized by increased inflammatory markers [C-reactive protein, interleukin (IL)-6], anemia (Hb < 12 g/dL), or low serum albumin (< 3.2 g/dL).^5^ As heart failure is an inflammatory disease, Anker et al.^6^ proposed that cardiac cachexia should be diagnosed when body weight loss is > 6% regardless of other criteria and in the absence of other severe diseases. More recently, investigators have used a body weight loss cutoff > 5% to characterize cardiac cachexia.^7,8^ It should be pointed out that cachexia is different from malnutrition or anorexia, which can both easily be reversed with adequate nutrition.^5^ Currently, several biomarkers have been studied to help diagnose cardiac cachexia.^9^ Muscle wasting is an important component of cachexia. It often precedes cachexia development and may also predict poor outcome in heart failure.^10^ Differently from cachexia, muscle loss diagnosis depends on the laboratory evaluation of muscle mass, such as dual energy X-ray absorptiometry (DEXA), computed tomography and magnetic resonance imaging.^11^ Muscle wasting may also be suggested by poor performance during spiroergometry, 6-min walking test, gait speed, or handgrip strength.^11^

The importance of cachexia in heart failure prognosis became more evident after the description of the reverse epidemiology of obesity in this condition. In healthy people, increased body mass index is associated with an elevated risk of developing cardiovascular disease. However, body mass index was positively correlated with survival in heart failure patients.^12^ In a meta-analysis of nine observational studies, mortality was lower in overweight and obese heart failure patients.^13^ The mechanisms involved in both the obesity paradox and the cachexia-induced worse prognosis are not completely clear.^14^

Cardiac cachexia prevalence varies between 8 and 42% according to cachexia definition and the study population.^6,7,15^ Anker et al.^6^ observed that 34% of heart failure outpatients had a ≥ 6% body weight loss during 48 months of follow-up. More recently, in optimally-treated nondiabetic outpatients, a > 5% body weight loss was observed in 10.5%.^7^

The etiology of heart failure-associated cachexia is multifactorial and the underlying pathophysiological mechanisms are not well established.^16^ Important factors include food intake reduction, gastrointestinal abnormalities, immunological and neurohormonal activation and an imbalance between anabolic and catabolic processes.^16,17^

## Clinical consequences of cachexia

The clinical consequences of cachexia depend on both weight loss and systemic inflammation, which accompany cachexia development. Severe body weight loss, even in the absence of systemic inflammation, is associated with deleterious effects on most organs and systems. Tissue loss from three compartments, lean tissue, fat mass, and bones, is usually found.^7^ In skeletal muscles, an imbalance between protein synthesis and breakdown leads to molecular changes and muscle atrophy, with decreased strength and daily activity impairment.^18-23^

The cardiac consequences of cachexia have been studied in heart disease-free conditions, such as cancer and undernutrition.^24-30^ In cachectic individuals, left ventricular mass correlated with lean body mass, showing that the heart is subjected to similar consequences to those in lean tissue during cachexia.^31^ In experimental animals, cancer cachexia induced cardiac dysfunction and molecular changes characteristic of the pathologic remodeling process with reduced anabolic pathway signaling.^24,25^ We observed that severe food restriction induces mild ultrastructural, morphological, and functional changes in normal rat hearts, which are exacerbated by hemodynamic overload in hypertensive rats.^32-37^ Therefore, the occurrence of cachexia can further impair cardiac changes and heart failure in a fatal vicious cycle. Cachexia can also exacerbate heart failure-associated anemia and gastrointestinal changes.^38^

## Cachexia prevention and treatment

As cardiac cachexia is multifactorial, it has been difficult to develop a specific therapy for its prevention and treatment.^11^ Since skeletal muscle wasting can precede cachexia, preventive strategies have been largely directed towards muscle mass preservation.^39^ Different options have been described, mostly evaluated in small clinical studies or experimental settings. These include nutritional support, neurohormonal blockade, reduced intestinal bacterial translocation, anemia and iron deficiency treatment, appetite stimulants, immunomodulatory agents, anabolic hormones, and physical exercise regimes (Table 1).^11^ Currently, nonpharmacological therapy such as nutritional support and physical exercise has been considered as the basis for cachexia prevention and treatment.^40^

**Table 1:** Cardiac cachexia: perspectives for prevention and treatment

**Nonpharmacological approach**
Nutritional support
Physical exercise

**Drug therapy approach**
*Treatment clinically useful*
Neurohormonal blockade
Reduction in intestinal bacterial translocation by peripheral edema control
Anemia and iron deficiency correction

*Experimental use only*
Supplementation of essential amino acids
Supplementation of branched-chain amino acids
Appetite stimulants
Immunomodulatory agents (pentoxyphylline, thalidomide, statins, methotrexate, N-acetylcysteine, T-cell activation inhibitors, chemokine antagonists, interleukin-10, interleukin-1 receptor antagonists)
Anabolic hormones (testosterone, growth hormone release-inducing, growth hormone)
Several mechanisms: myostatin inhibitors and antagonists, bortezomide, lipopolysaccharide bioactivity inhibitors, and melanocortin blockers

### Nutritional support

Non-obese patients with stable heart failure often have inadequate food intake.^41^ Therefore, nutritional support is recommended to obtain and maintain a body weight within or a little below the normal range without edema. Currently, there is no specific recommendation for protein and energy intake. The ingestion of 35 kcal/kg/day was shown to be safe and effective in increasing lean mass in heart failure patients.^42^ Some authors have recommended a caloric intake of at least 31.8 kcal/kg/day.^41^ Nutritional support should be started with small amounts and slowly increased until desired body weight is reached. Excess energy intake increases catecholamine and insulin plasma concentrations causing physiological stress. An increase in insulin levels induces renal sodium and water reabsorption and may decompensate cardiac failure. Therefore, patients should be advised to evaluate their body weight daily and tailor diuretic therapy. Protein intake should follow recommendations for healthy people and may be increased in cases of protein loss by intestinal malabsorption or nephropathy. However, a small trial showed that high-caloric protein-rich oral nutritional supplement improved body weight and reduced inflammatory markers.^43^ Sodium intake depends on heart failure functional class, being more restricted (0.5 to 2 g/day) in severe cases; this is when patients need to be educated on food sodium content. Chronic and vigorous use of diuretics can deplete potassium and magnesium levels. With increased carbohydrates and amino acids intake and increased insulin levels, there is a shift in potassium, magnesium and phosphorus from extracellular to intracellular compartments, thus decreasing plasma concentrations of these electrolytes which can induce cardiac arrhythmias and sudden death.

There is no specific recommendation for micronutrients in heart failure. Reduced food intake and chronic use of diuretics can cause water-soluble vitamin deficiency. Thiamine needs particular attention as a deficiency may impair cardiac function.^44^ Intestinal malabsorption can reduce plasma levels of the soluble vitamins A, D, E, and K.^44^ As liver congestion and ascites cause food intake intolerance, meals should be frequent and small. It should be stressed that, despite the importance of nutritional support, it has not been established whether adequate protein-energy intake can reverse nutritional status in chronic heart failure.^45^ Furthermore, increased food intake may compensate some weight loss, but it can change tissue distribution towards increased fat mass, particularly when muscle loss is present.^46^ Therefore, to preserve or recover muscle mass, nutritional support should be combined with physical exercise.

Recent small studies have suggested that alterations in specific diet components may be useful in cardiac cachexia. For example, supplements of essential amino acids improved nutritional and metabolic status in most muscle-depleted heart failure patients.^45^ Supplementation of branched-chain amino acids, which consist of leucine, isoleucine, and valine, preserved body weight, skeletal muscle mass, and cardiac function in rats;^47^ however, it failed to benefit heart failure patients.^48^

### Neurohormonal activation blockade

Chronic heart failure is characterized by sustained cardiac and systemic activation of the renin-angiotensin-aldosterone and adrenergic nervous systems which, in the long term, impairs ventricular remodeling. Therefore, blockade of these systems is recommended for all heart failure patients with reduced ejection fraction.^2,49^ The heart failure control with neurohormonal blockade can reverse cachexia independently of nutritional support.

However, neurohormonal activation is also directly involved in skeletal muscle atrophy. The effects of angiotensin II can be prevented by angiotensin-converting enzyme inhibitors (ACEi) and angiotensin 1 receptor blockers. More recently, angiotensin II has been shown to play a role in cachexia and skeletal muscle wasting through different mechanisms such as increased oxidative stress and protein breakdown; impaired energy balance; reduced appetite via alteration in orexigenic/anorexigenic neuropeptides in the hypothalamus; and inhibition of satellite cell function and muscle regeneration.^50,51^ Administration of ACEi enalapril decreased the risk of weight loss in heart failure patients.^6^ One may argue that as angiotensin II antagonism improves cardiac remodeling and ventricular function, it would also reduce the risk for cachexia development. Thus, neurohormonal blockade was also examined in cancer cachexia. In tumor-bearing rats, angiotensin and aldosterone antagonism as well as adrenergic nervous system blockade attenuated body weight and lean mass loss.^25^ In a phase III clinical trial, ACEi imidapril prevented weight loss in patients with cachexia caused by non-small cell lung cancer and colorectal cancer but not by pancreatic cancer. When data were combined however, weight loss prevention did not reach statistical significance.^51^ Future studies are needed to elucidate the role of neurohormonal blockade in different causes of cachexia.

### Reduction in intestinal bacterial translocation

Heart failure patients with peripheral edema present increased intestine wall thickness, which suggests bowel wall edema.^38^ Among echocardiographic parameters, the combination of right ventricular dysfunction and elevated right atrial pressure provided the best discrimination between cachectic and non-cachectic patients.^52^ Furthermore, cardiac cachexia was associated with intestinal congestion irrespective of heart failure stage and cardiac function.^52^ Heart failure patients also have a reduction in intestinal blood flow and an increase in juxtamucosal bacterial growth.^53^ These abnormalities lead to intestinal bacterial translocation and systemic immune activation.^53,54^ Bacterial endotoxins, also known as lipopolysaccharides, are potent inducers of pro-inflammatory substances such as tumor necrosis factor (TNF)-α. As intensive diuretic therapy normalized increased endotoxin levels in heart failure patients with peripheral edema,^54^ patients should have as little edema as possible by using one or a combination of diuretics.^49^ Despite experimental studies showing antibiotic therapy decreases intestinal bacterial translocation, it is not established whether microflora modulation is safe or useful in reducing systemic immune activation in heart failure. Therefore, this approach is not currently recommended.^55^

### Anemia and iron deficiency treatment

The prevalence of anemia in heart failure ranges from 4% to 55%, according to study population and anemia definition.^56^ Anemia is associated with increased mortality, hospitalization, and impaired quality of life.^57^ Anemia etiology in heart failure is multifactorial. Iron deficiency is present in about half of heart failure patients, independent of the presence of anemia.^56^ Both anemia and iron deficiency are associated with reduced exercise tolerance.^58^ As decreased exercise capacity is related to a reduced skeletal muscle mass, anemia and iron deficiency may be involved in cachexia development. Diagnostic evaluation for reversible causes of anemia and subsequent treatment is appropriate in all patients. Currently, several heart associations suggest that iron deficiency should be routinely checked in all heart failure patients and corrected if present.^56^ Intravenous iron preparations are safe and effective in treating iron deficiency;^58^ little information is available on the effectiveness of oral iron.^56^ Intravenous iron correction of iron deficiency was associated with improved functional status.^59^ As erythropoiesis stimulating agent darbepoetin alpha failed to improve clinical outcomes in heart failure patients with mild-to-moderate anemia,^60^ this class of drug is not recommended for treating heart failure-associated anemia.

### Perspectives for future treatment of cachexia

Several pharmacological agents have been tested in experimental and clinical settings for preventing and treating cardiac cachexia. However, they currently represent perspectives for the future and are not recommended for clinical use.

Appetite loss is a common finding in cardiac cachexia and its origin is multifactorial.^15^ Although appetite stimulants such as megestrol acetate have been used in other cachectic conditions, they are not approved for cardiac cachexia.

As previously stated, chronic cardiac failure is followed by immunologic activation, which plays an important role in cachexia development. Therefore, several immunomodulatory agents have been tested in heart failure. Tumor necrosis factor (TNF)-α antagonists etanercept and infliximab were tested in large clinical trials with neutral or negative results.^61^ Pentoxyphylline and thalidomide, also considered immunomodulatory agents, were used in small trials with neutral or favorable results.^62,63^ Other immunomodulatory drugs such as statins, methotrexate, N-acetylcysteine, T-cell activation inhibitors, chemokine antagonists, IL-10, and IL-1 receptor antagonists have been tested in experimental studies.^19,64^

Anabolic hormones have also been examined to preserve and/or increase muscle mass. Testosterone levels decrease with age; this phenomenon being faster in heart failure men than in their healthy male counterparts.^65^ Low concentration of testosterone was related to increased risk of death, independently of left ventricular function or functional capacity.^39,65^ In skeletal muscle, testosterone increases protein synthesis, reduces protein breakdown, and stimulates proliferation and differentiation of satellite cells, thus increasing muscle mass and strength, and improving exercise capacity.^39^ Therefore, androgen deficiency may be involved in the imbalance between anabolic and catabolic processes and contribute to heart failure-induced muscle wasting and cachexia.^65^ Testosterone supplementation was evaluated in small randomized double-blind studies including elderly men^66^ and women^67^ with heart failure. As testosterone improved functional capacity and muscle strength, it was hypothesized that it could be safe and useful in heart failure and cardiac cachexia.

Growth hormone release-inducing (Ghrelin) increases adiposity and food intake by modulating neural circuits that control food intake, energy expenditure, and reward.^68^ Ghrelin has been evaluated in small trials in different cachectic conditions.^15^ In heart failure, repeated Ghrelin administration improved exercise capacity and muscle wasting, suggesting that Ghrelin and its receptor agonist anamorelin may be an attractive approach for future investigation.^68,69^ Growth hormone (GH) also have the potential to improve muscle mass and functional capacity.^70^ However, as their effects are not completely established in heart failure patients,^71,72^ additional research is needed to clarify the role of GH in cardiac failure and cachexia.

Currently, several drugs such as myostatin inhibitors and antagonists, bortezomide (an ubiquitin-proteasome route inhibitor), lipopolysaccharide bioactivity inhibitors, and melanocortin blockers have been investigated with the purpose of preserving and/or increasing muscle mass in cardiac cachexia.^9,20,22^

### Physical Exercise

Physical exercise is the most promising option for treating muscle wasting in several diseases. Current heart failure guidelines strongly recommend regular physical exercise for stable patients to prevent and/or attenuate cardiac remodeling and skeletal muscle alterations.^2,49,73^ Clinical and experimental studies have shown that aerobic exercise improves cardiac remodeling and ventricular function, and increases functional capacity and quality of life.^74-76^ In skeletal muscle, exercise training reduces oxidative stress, activation of the ubiquitin-proteasome system, expression of myostatin and proinflammatory cytokines, sympathetic nerve activity and peripheral vasoconstriction, reestablishes expression of proteins involved in sarcoplasmic calcium handling, and prevents capillary rarefaction and atrophy.^77-79^

Other exercise modalities have also shown promising results in heart failure. For example, a resistance exercise program improved functional capacity^48^ and a combination of hydrotherapy with endurance training improved exercise tolerance and hemodynamic profile of heart failure patients.^81^ Additionally, high-intensity aerobic exercise was safe and superior to moderate-intensity aerobic training in increasing maximal oxygen consumption.^82^ Therefore, additional studies are needed to establish the best training protocol relating to exercise type, intensity, duration, and frequency to improve outcomes in cardiac cachexia.

## Conclusion

Cachexia plays an important role in morbidity and mortality in heart failure patients. Understanding the pathophysiological mechanisms that cause cachexia is an essential step to developing pharmacological and non-pharmacological strategies aimed at effectively preventing and treating heart failure-induced cachexia before significant body weight and muscle wasting occurs. Currently, nonpharmacological therapy such as nutritional support and physical exercise are the basis for cachexia prevention and treatment.
